# On the possible role of nonreproductive traits for the evolution of unisexuality: Life‐history variation among males, females, and hermaphrodites in *Opuntia robusta* (Cactaceae)

**DOI:** 10.1002/ece3.4217

**Published:** 2018-06-11

**Authors:** Rafael F. del Castillo, Sonia Trujillo‐Argueta

**Affiliations:** ^1^ CIIDIR Oaxaca Instituto Politécnico Nacional Santa Cruz Xoxocotlán México

**Keywords:** clonal growth, dioecy, growth rates, herbivory, meta‐analysis, secondary sex characters

## Abstract

In angiosperms, dioecy has arisen in 871–5,000 independent events, distributed in approximately 43% of the flowering families. The reproductive superiority of unisexuals has been the favorite explanation for the evolution of separate sexes. However, in several instances, the observed reproductive performance of unisexuals, if any, does not seem to compensate for the loss of one of the sex functions. The involvement of fitness components not directly associated with reproduction is a plausible hypothesis that has received little attention. Life‐history traits recently recognized as predictors of plant performance were compared among males, females, and hermaphrodites of a rare trioecious *Opuntia robusta* population in the field, using the cladode as the study unit. Cladode mortality by domestic herbivores was common and higher in females and hermaphrodites than in males. Males, females, or both displayed lower shrinkage and higher rates of survival, growth, and reproductive frequency than hermaphrodites. Unisexuals simultaneously outperformed hermaphrodites in demographic traits known to compete for common limiting resources, such as the acceleration of reproductive maturation (progenesis) and survival. A meta‐analysis combining the outcomes of each of the analyzed life‐history traits revealed a tendency of males (*d*_++_ = 1.03) and females (*d*_++_ = 0.93) to outperform hermaphrodites in presumably costly demographic options. Clonality is induced by human or domestic animal plant sectioning; and males and females highly exceeded hermaphrodites in their clonality potential by a factor of 8.3 and 5.3, respectively. The performances of unisexuals in the analyzed life‐history traits may enhance their reproductive potential in the long run and their clonality potential and could explain the observed increase of unisexuality in the population. Life‐history traits can be crucial for the evolution of unisexuality, but their impact appears to be habitat specific and may involve broad ontogenetic changes.

## 1 INTRODUCTION

The presentation of combined or separate sexes in a single individual is one of the most contrasting and intriguing variations of sexual expression in plants. In angiosperms, hermaphroditism is likely the ancestral condition from which 871 to 5,000 estimated independent evolutionary events have led to dioecy (populations with male and female individuals) (Renner, 2014). Selection is often believed to explain this transition, in particular the advantages of canalizing resources to a single‐sex function instead of two and the reputed role of unisexuals to enforce outbreeding by suppressing selfing and thereby reducing inbreeding depression risks (see Charlesworth, 2006). Nonetheless, the reproductive advantage of unisexuals over hermaphrodites is not universal, or such advantage does not always seem to compensate the full sterility of the nonfunctional sex (e.g., *Cucurbita foetidissima*, Kohn, 1989; *Trifolium hirtum,* Molina‐Freaner & Jain, 1992; *Phacelia dubia* del Castillo, 1993; *Pachycereus pringlei*, Fleming, Maurice, Buchmann, & Tuttle, 1994; *Daphne laureola*, Alonso & Herrera, 2001; *Kallstroemia grandiflora,* Cuevas‐Garcia, Marquez, Dominguez, & Molina‐Freaner, 2005; *Opuntia robusta*, del Castillo & Trujillo‐Argueta, 2009; Janczur et al., 2014; *Dianthus sylvestris* Collin & Shykoff, 2010; and *Geranium maculatum*, Van Etten, Deen, Hamrick, & Chang, 2014; see also Dufay & Billard, 2012 for a review on gynodioecious species). One plausible explanation is that unisexuality evolves indirectly via selection on correlated nonreproductive traits.

Sex‐specific traits not directly associated with reproduction are common in dioecious plant species (Barrett & Hough, 2013; Geber, Dawson, & Delph, 1999; Lloyd & Webb, 1977). Some of these traits are crucial for fitness and might influence the evolution of separate sexes. Nonreproductive differences between males and females appear to result from trade‐offs between reproduction and other vital functions. Indeed, the commonly observed lower growth rate of females compared to that of males has been explained by the usually higher cost of reproduction incurred by females (see Barrett & Hough, 2013; Delph, 1999; Obeso, 2002). Gender‐specific differences should be even more pronounced between unisexuals and hermaphrodites as the latter allocate resources to both sexual functions in contrast to one. However, they have received little attention.

Evolutionary shifts from hermaphroditism to unisexuality probably affect fitness through alterations in life history. Organisms develop life‐history strategies involving trade‐offs among plant functions that may account for their performance in a particular habitat. A recent worldwide study, based on demographic data of 418 plant species, identified two major life‐history strategies that promote population persistence (Salguero‐Gomez et al., 2016). In a principal components (PC) analysis of these data, the first PC axis identified increases in survival and longevity with reductions in growth and recruitment. The second PC axis identified that reproductive frequency and the average number of recruits produced during the life expectancy of an individual in the population was negatively associated with retrogressive growth (shrinkage). Indeed, some plants can increase survival in adverse conditions by shrinking (Salguero‐Gómez & Casper, 2010). In addition, the modular nature of plants allows them to propagate clonally and thus to increase fitness through genet persistence and seed production (Pan & Price, 2002). Therefore, survival, growth, shrinkage, reproduction, and cloning are likely the minimum set of features required for analyzing the fitness consequences of having one or both functional sexes in a single individual.

Studies addressing gender‐specific differences in life‐history traits can help elucidate the evolutionary forces favoring contrasting patterns of sex allocation. Sexual polymorphic populations with male, female, and hermaphroditic individuals (trioecy) are particularly convenient for this task. First, the existence of three genders allows comparing individuals with three contrasting levels of sex allocation: only the male function, only the female function, and both sexual functions. Second, such studies can be conducted on the same population with individuals of different genders but otherwise relatively similar phenotypes, eliminating the phylogenetic noise resulting from comparing individuals of different species (see Dawson & Geber, 1999; Delph, 1999). Third, these studies can be conducted under a common and natural environment for the three genders. Studies comparing the ecology of unisexuals and hermaphrodites have focused mostly on reproductive allocation (e.g., Ashman, 1999; Case & Barrett, 2001; Perry, Pannell, & Dorken, 2012; Van Drunen & Dorken, 2012), pollination (e.g., Ashman & Stanton, 1991; Ramula, Toivonen, & Mutikainen, 2007), or herbivory (e.g., Sánchez‐Vilas & Pannell, 2011). Only few studies have compared vegetative performance, and these were based on limited selected traits (e.g., Case & Barrett, 2001; Zunzunegui et al., 2006). We are not aware of any study exploring all of the essential life‐history traits found by Salguero‐Gomez et al. (2016) in hermaphrodites, males, and females.

One of the rare species of angiosperms with trioecious populations is *Opuntia robusta* (Cactaceae). A previous study showed that under natural conditions, *O. robusta* hermaphrodites reproductively outperform unisexuals because limited pollination services prevent unisexuals from reaching their full reproductive potential. Males produced on average 6.5‐fold more pollen than hermaphrodites, but natural fruit production in females was only 41.5% that of hermaphrodites, despite flower and ovule production in females and hermaphrodites are similar (del Castillo & Trujillo‐Argueta, 2009). Under pollen‐limited conditions, hermaphrodites can reproductively outperform females because automatic selfing reduced their dependency on external pollination, inbreeding depression was undetectable, and prior selfing reduced outcrossing opportunities from conspecific and heterospecific sources. Furthermore, because of the high pollen production (pollen/ovule = 526), hermaphrodites' pollen can be an important source of genes (del Castillo & Trujillo‐Argueta, 2009). Under such conditions, theory predicts the breakdown of dioecy and the appearance of males with partial female functions (Crossman & Charlesworth, 2014). This hypothesis is supported in Cactaceae by the detection of “weakly hermaphroditic” morphs in *Consolea* spp.*,* a close relative to *Opuntia* spp. (Strittmatter, Hickey, & Negrón‐Ortiz, 2008). However, all *O. robusta* unisexuals examined were fully sterile in their nonfunctional sex, whereas hermaphrodites were fully fertile for both functions (del Castillo & Trujillo‐Argueta, 2009). Furthermore, in the studied population, the proportion of hermaphrodites dropped from 77.34% (with 11.05% males and 11.61% females; *n *=* *353) in 1984 to 49.44% (with 26.39% males and 24.16% females; *n *=* *538) in 2014 (R.F. del Castillo, *unpublished data*). Maurice and Fleming (1995) in a simulation study showed that pollen limitation can increase the probabilities of the stability of trioecy and the maintenance of hermaphrodites at the expense of unisexual types, but the increases in frequency of *O. robusta* unisexuals*,* given their observed reproductive performance are not consistent which such predictions.

**Figure 1:**
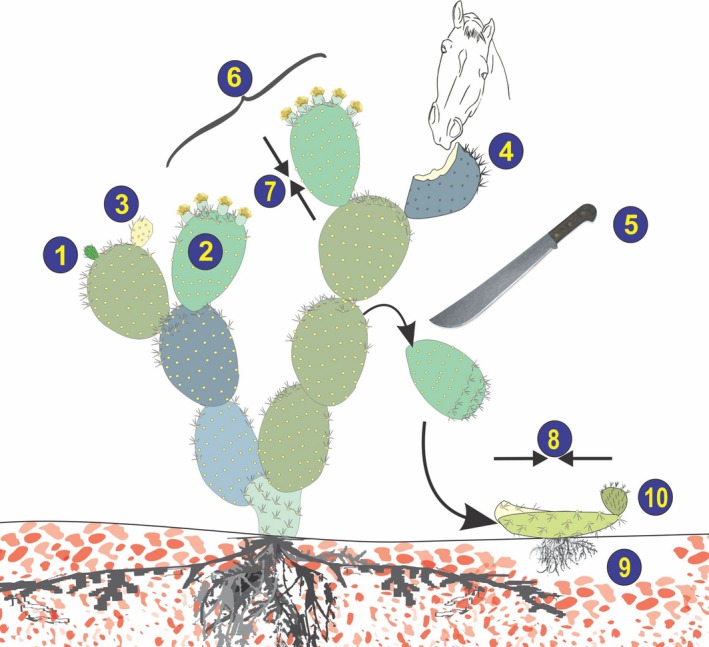
Diagram of some of the life‐history characteristics of *Opuntia robusta* investigated in the present study. (a) Cladode survival from bud stage (1) to adult size (2). (b) Causes of cladode loss from the mother plant, such as yellowing (3), mechanical removal of the cladode or parts or it by large domestic herbivores (4), or detaching, usually conducted by ranchers (5). (c) Frequency of flowering, estimated by the proportion of terminal cladodes with flowers (6). Note here than some terminal cladodes were not reproductive, such as the cladode with vegetative buds on the left, one partially eaten cladode (on the top right), and one artificially detached cladode (on the center left). (d) Retrogressive growth estimated in rooted plants (7), or in detached cladodes (8). (e) Clonality, estimated by the proportion of detached cladodes that set roots (9), and produce new vegetative shoots (10)

One possible explanation for such unexpected results is that unisexuals outperform hermaphrodites in other fitness components by expending less in reproduction in a given bout of reproduction. Such savings may translate into higher survival of their ramets and more opportunities for reproduction in the future. In the present study, we compare the life‐history of all genders in sexually polymorphic *O. robusta* to explore this possibility. We extend some of Delph's (1999) predictions for dioecious plants to hypothesize that, within trioecious populations, males, which do not invest in ovules and fruits, might on average outperform females, which produce ovules and fruits but not pollen, in presumably costly life‐history parameters. Namely males will have higher rates of ramet survival, frequency of reproduction, clonality, and growth, and lower rates of retrogressive growth (Figure 1). Similarly, in such parameters, females should outperform hermaphrodites by being more pollen‐limited than hermaphrodites in some populations and produce only ovules and fruits, but no pollen (see del Castillo & Trujillo‐Argueta, 2009).

## 2 MATERIALS AND METHODS

### 2.1 Study system

*Opuntia robusta* (Cactaceae) is a sexually polymorphic species with hermaphroditic, dioecious, and trioecious populations (del Castillo & Trujillo‐Argueta, 2009). Some evidence suggests that *O. robusta* gender is determined by nuclear genes: (a) in sexually polymorphic populations, male: female ratio is not significantly different from 1:1; (b) males are not spatially segregated from females; (c) plant size does not appear to influence sex expression; and (d) gender appears to be constant throughout the life span of the individual (del Castillo, 1986; del Castillo & Trujillo‐Argueta, 2009). In the dioecious and closely related *O. stenopetala*, male sterility takes place by anther abortion caused by premature cell death and DNA fragmentation (Flores‐Renteria et al., 2013). *Opuntia robusta* has a modular structure in which each unit (ramet), botanically known as cladode, has a determinate growth completed in one season. If detached, cladodes can exist independently, generating a new plant. Thanks to this modular nature, we can gain knowledge of the life‐history of *O. robusta* by focusing on the demography of its cladodes. Stem cells are regularly distributed on the surface of the cladodes in structures called areoles. With proper cues, areoles can develop spines, glochidia, roots, rudimentary leaves, stems (cladodes), or flowers. Cladode growth initiates from a bud generated by one areola and concludes during one growing season. Usually, only one areola per 1‐year‐old cladode produces a vegetative bud, which develops into a new full‐sized cladode. The present study focuses on the gender‐associated life‐history polymorphism of a trioecious population of *O. robusta* using the cladode as a unit of study.

In *O. robusta,* reproductive allocation is expected to be higher in hermaphrodites than in females and in females than in males for the following reasons. First, hermaphrodites produce pollen and ovules, whereas males only produce pollen and females only produce ovules (del Castillo & Trujillo‐Argueta, 2009; see also Janczur et al., 2014). Second, flowers of hermaphrodites are larger and produce higher quantities of nectar than those of males and females (del Castillo & González‐Espinosa, 1988). Third, the three genders bloom from early spring to late May. After this month, males have virtually no further reproductive expenses until the next growing season. However, most of fruiting and cladode elongation starts in late May or early June, just after blooming (cf., del Castillo & González‐Espinosa, 1988; González‐Espinosa & Quintana‐Ascencio, 1986; this study). Thus, while males are exempt of reproductive costs during most of the time at which cladode growth takes place, fruiting should affect cladode elongation in females and hermaphrodites given the synchrony of both processes. Indeed, the timing of investment has been found to be as critical for plants as the magnitude of the investment (Lloyd, 1980; Meagher & Delph, 2001). Additional savings in reproduction are also expected in females, as these are more pollen‐limited than hermaphrodites in some populations, and produce fewer seeds and fruits per reproductive bout (del Castillo & Trujillo‐Argueta, 2009). Given that fruits and cladodes are metabolic sinks likely competing for the same resources, females are expected to produce more cladodes compared with hermaphrodites when investing less in fruit production under pollen‐limited conditions (see Lloyd, 1980). Seed biomass is a good indicator of female reproductive costs in *Opuntia* spp. and is highly correlated with fruit biomass (Inglese, Barbera, & Carimi, 1994; López‐Palacios et al., 2015).

### 2.2 Study site

Our study was conducted in the semiarid highlands of Santa Rosa and neighboring areas of Central Mexico, San Luis Potosí State (see del Castillo & Trujillo‐Argueta, 2009, for a detailed description of the study area). The study area is mostly a semidesert scrub used as a rangeland for cattle and horses, dominated by *Opuntia* spp*., Prosopis* spp., *Aloysia gratissima*, and *Zaluzania augusta*. *Opuntia robusta* is used as fodder, although the spines partially deter plant consumption. Local ranchers commonly detach a section of the plant off for rooting and sprouting. The newly produced shoots, if any, are more palatable than tough adult cladodes: they are juicier, tender, and with softer spines. Some of these cladodes escape or survive herbivory, developing into a new plant.

### 2.3 Cladode survival and sources of cladode loss

We monitored the survival of 298 cladodes from 156 plants (29 females, 96 hermaphrodites, and 31 males) for 8 months, that is, from the bud stage and until they perished or up to 3 months after reaching the adult stage. The plants were monitored at 10–15 day intervals before and during the exponential growth phase, which took place in late spring or summer. During fall and winter, cladodes attained maximum size and they were monitored at 20–30 day intervals. Three kinds of cladode losses from the mother plant were identified: (a) mechanical damage, in which all or part of the cladode was eaten by a large herbivore, usually horses or cattle; (b) detaching or sectioning by ranchers, easily distinguished from those by herbivores by the cut being straight instead of curvy; and (c) dying by nonmechanical causes, such as phytophagous insects, photoinhibition, or desiccation, usually at early stages, in which case the cladode dies intact turning dry or yellow. We compared these types of cladode loss among genders at the end of the monitoring period using a logistic model for categorical data (generalized logit [alive/dead]) and maximum‐likelihood parameter estimation with the procedure CATMOD within SAS software (ver. 9.12; SAS Institute Inc., Cary, NC, USA). Plant gender and the cladode size at which the cladode was last recorded as living were the dependent variables. Cladode size, estimated as the log of the product of width and length, was expected to affect cladode survival. We considered data from cladodes of distinct individuals as independent and data from cladodes monitored within a single plant as dependent. To discriminate between these two kinds of observations, cladodes were nested within plants in the cross‐classification of the rows within the contingency table of the model using the population statement of CATMOD. An additional advantage of this procedure is its built‐in goodness‐of‐fit test: the residual chi‐square. The major sources of cladode losses from the mother plant were tested for gender‐association using a standard categorical analysis of variance with the logit link and the CATMOD procedure.

### 2.4 Cladode growth rates

The growth pattern of cladodes resembles a sigmoidal curve with its three parameters. The initial lag phase with low growth rates and a time length λ is followed by an exponential phase during which the maximum growth rate, μ, is attained at the inflexion point of the growth curve. Then, the growth rate decreases, and the cladode reaches its maximum size *A*. We monitored the size of randomly selected vegetative buds until cladodes reached adult size at least five times (mean 9.4, maximum 13 times) during the growth period. Because cladode mortality was high, newly produced vegetative buds had to be monitored to replace those that perished. Data were obtained from 115 cladodes: 52 cladodes from 49 hermaphrodite plants, 30 cladodes from 19 male plants, and 33 cladodes from 21 female plants that reached the maximum size. The log of the product of cladode length and width was used as the size estimator. The growth of each cladode was fitted using the parametric Gompertz and logistic models (see Kahm, Hasenbrink, Lichtenberg‐Fraté, Ludwig, & Kechisko, 2010 for formulae). For each cladode, the best parametric model was selected based on the Akaike criterion (AIC), and *A*, λ, and μ were estimated. Model fitting and selection was conducted using the grofit package (gc function; Kahm et al., 2010) of R (R Core Team, 2014). The gcBootSpline procedure within this package was used to obtain another set of parameter estimates using a model‐free method (cubic fitting spline model) for the growth curve of each of the monitored cladodes. This procedure was useful in the few cases in which the parametric fit could not be achieved or was poor. Based on the cubic fitting spline model and the best parametric model for the growth of each cladode, the best set of parameters was selected from the model with the lowest 90% confidence interval, which was obtained from a standard bootstrap procedure with 100 samples using the grofit package. Growth parameters were compared among cladodes using ANOVAs, with the average of each parameter obtained from all the monitored cladodes in a given plant as data entries. Because each average was derived from a different number of cladodes, data obtained from a larger sample size was given a higher weight using the weighted‐least‐squares procedure.

### 2.5 Retrogressive growth (shrinkage)

After attaining their maximum size, some of the cladodes monitored during growth analysis displayed size reductions. We investigated if such reductions were gender‐associated by analyzing shrinkage rates in cladodes from rooted plants and in cladodes detached from their mother plants. In cladodes from rooted plants, we continued to measure their size during 3–4 months after they reached their maximum size and 2–4 times during that time interval; the minimum size observed during this period was then compared to the maximum size observed for each cladode. The relative shrinkage rate was expressed as the difference between these two measurements divided by the number of elapsed days and the initial size. The data were statistically analyzed using ANOVA as described in the section above. In detached cladodes, shrinkage was estimated as the rate of weight loss of terminal cladodes manually detached from different plants (Terminal cladodes are those cladodes that do not have any full‐sized cladode on top and are usually one‐year old, Figure 1). For this task, 33 adult cladodes from different plants belonging to each gender were separated from the mother plant exactly at the level of the joint using a sharp knife and set horizontally in the field in random groups of three (one hermaphrodite, one male, and one female). The cladodes were weighed immediately after being detached and 71–79 days later, before they started rooting. The relative rates of weight loss per day and initial weight were compared using a one‐way ANOVA.

### 2.6 Flowering frequency

Most vegetative and reproductive buds are produced in terminal cladodes. Thus, we determined the number of terminal cladodes with flowers (R) and the total number of terminal cladodes (T) in 296 randomly selected reproductive individuals (47 females, 177 hermaphrodites, and 72 males) to estimate the ratio of terminal cladodes with flowers over the total number of terminal cladodes here defined as flowering frequency (R/T). We tested if R/T varied among genders with a standard ANOVA.

### 2.7 Vegetative propagation potential (clonality)

To test gender differences in clonality, we examined the fraction of the detached cladodes used for cladode shrinkage that rooted and the fraction that produced vegetative shoots (30 male, 26 female, and 24 hermaphrodite cladodes; some cladodes disappeared for unknown reasons). We monitored cladodes for 9 months. After this time, unrooted cladodes perished, probably because of desiccation. To compare rooting and sprouting rates among the different genders, we conducted Boschloo's tests between all pairs of gender combinations using the Test package within R (Calhoun, 2015). This test is more powerful than traditional alternatives, it is suitable for small sample sizes and performs unconditional exact tests of 2 × 2 contingency tables with fixed marginal totals for only one criterion (gender, in our case; Calhoun, 2015). Unless otherwise stated, all statistical tests performed were two tailed.

### 2.8 Combined gender differences in vegetative performance

Meta‐analysis techniques were used to compile and quantitatively synthesize gender‐associated differences in life‐history traits. Meta‐analysis provides an overall measurement of the tested effect and reduces the likelihood of a type II error (i.e., falsely assuming no effect when there is an effect) (Hunter & Schmidt, 2004).

We conducted pairwise comparisons for all life‐history traits analyzed to test the hypothesis that the gender that presumably expends less in reproduction will display a higher overall performance in life‐history parameters that are expected to be more costly. Such gender would exhibit: (a) higher cladode survival; (b) shorter lag phase during cladode growth; (c) higher maximum cladode growth rate; (d) larger maximum cladode size; more frequent (e) rooting and (f) budding of detached cladodes; (g) higher flowering frequency in terminal cladodes; and lower shrinkage in (h) attached and (i) detached cladodes. Thus, overall performance is expected to be higher in males (M) than in females (F) and hermaphrodites (H), and in females than in hermaphrodites. We tested such individual expectations in each of the life‐history traits using linear contrasts or in 2 × 2 contingency tables (clonality) using one‐tailed tests. As described below, we estimated the magnitude of effects in the traits analyzed considering each of the hypotheses, obtained indexes for the overall effect sizes, and combined individual *p*‐values to conduct a joint overall test of significance.

For each trait, we conducted pairwise gender comparisons based on the standardized mean difference, *d*, defined as: (*M*_*1*_ *–M*_*2*_) *J S* ^*−1*^, where *M*_*1*_ *–M*_*2*_ is the difference between the means obtained for a given trait in each of the two genders, *S* is the pooled standard deviation, and *J* is a correcting factor for small sample size bias (Hunter & Schmidt, 2004). We used the original standard error, *SE*, for estimations of *d*, following the Dunlop, Cortina, Vaslow, and Burke (1996) recommendation for correlated designs. For dichotomous variables, we calculated *SE* from the log odds ratio, *log(OR),* and the *z*‐values derived from the *p*‐values of the corresponding tests: *SE = log*_*e*_ *(OR)z* ^*−1*^. Overall size effects were estimated with weighted and unweighted averages. The weighted average (*d*_*++*_ *)* is the standard index used in meta‐analysis literature and, therefore, is convenient for comparative purposes. This index gives more weight to the *d* values with lower variance and is defined as:

$$d_{++}=\frac{\sum_{i=1}^{t}w_{i}d_{i}}{\sum_{i=1}^{t}w_{i}},$$

where *d*_*i*_ is the effect size of trait *i* between two given genders, *w*_*i*_ is the reciprocal of the sampling variance of trait *i* of the two genders, and the sum considers all analyzed traits *t*. The unweighted average (*u*) gives equal weight to all traits regardless their sampling variance. An effect size not significantly different from zero indicates there is no gender effect. As widely accepted in the literature, effect sizes of 0.2, 0.5, and 0.8 or greater indicate small, medium, or large effects, respectively, in the direction of the tested hypothesis. Similarly, −0.2, −0.5, or −0.8 or smaller indicate small, medium, or large effects but in the direction opposite to the tested hypothesis. Nonparametric percentile confidence intervals of 95% were calculated using the bootstrap package of R (version 2017.2, https://CRAN.R-/package=bootstrap). One‐tailed *p*‐values from each individual test were combined using the CombinePValue and the competitive.test (Dai, Leeder, & Cui, 2014) packages of R for all the hypotheses tested (i.e., overall performance in M > F, in F > H, and in M > H). The competitive.test performs a modified Fisher test for correlated data and tests if the *p‐*value vector, a vector with all the individual *p*‐values of the performed tests, is more significant than randomly selected *p*‐values (Dai et al., 2014). Compared with the confidence intervals of the effect sizes, this procedure provides an alternative significance test based on all individual *p*‐values of the tested hypothesis.

## 3 RESULTS

### 3.1 Cladode survival, herbivory, and other sources of mortality

**Figure 2:**
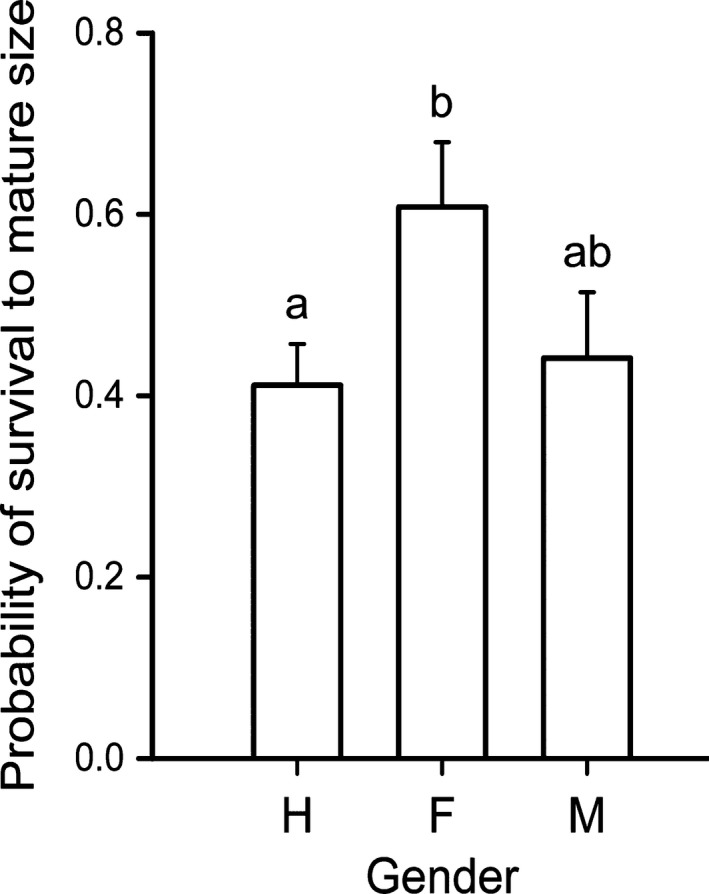
Probabilities of survival from bud to mature stage of cladodes of *Opuntia robusta* from hermaphrodite, female, and male in rooted plants. Bars sharing the same letter are not statistically different (*p* > 0.05)

Cladodes' survival probability was associated with plant gender ($\chi_{2}^{2}$ = 7.10, *p* = 0.026), and significantly increased with cladode size ($\chi_{1}^{2}$ = 37.04, *p* < 0.0001). The survival rate of cladodes of females was the highest among the three genders and significantly differ from those of hermaphrodites ($\chi_{1}^{2}$ = 6.96, *p* = 0.0084). The survival rate of cladodes of male plants was intermediate and not significantly different from those of females ($\chi_{1}^{2}$ = 2.58, *p* = 0.108) and hermaphrodites ($\chi_{1}^{2}$ = 1.09, *p* = 0.296; Figure 2). Size‐by‐gender interaction was not significant ($\chi_{2}^{2}$ = 5.03, *p* = 0.081), but a significant residual variance ($\chi_{150}^{2}$ = 191.55, *p* = 0.0124) indicated that other unknown factors are involved in cladode survival.

**Figure 3:**
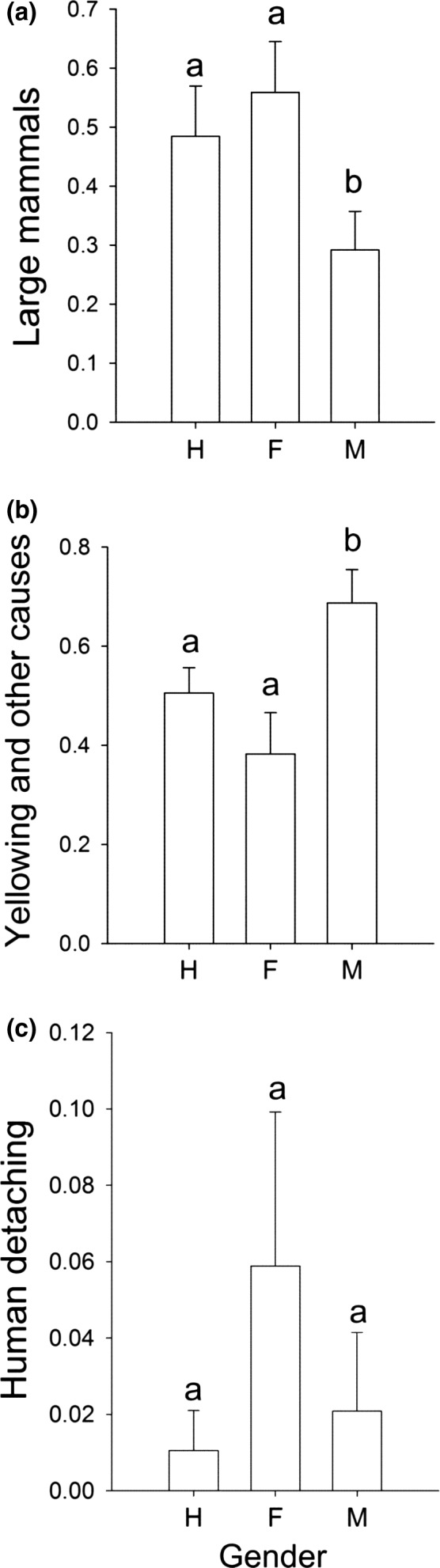
Types of cladode loss from the mother plant of *Opuntia robusta* by (a) consumption of big‐domestic mammals; (b) drying, yellowing, or phytophagous insects, in which case the cladode perish intact, no parts of the cladode were taken; and (c) sectioning inflicted by ranchers in Santa Rosa, San Luis Potosí, Mexico. Bars sharing the same letter in the same graph are not statistically different (*p* > 0.05)

Cladode mortality rates attributed to herbivory by large mammals differed significantly between males and females ($\chi_{1}^{2}$ = 5.74, *p* = 0.017) and between males and hermaphrodites ($\chi_{1}^{2}$ = 4.75, *p* = 0.029). Cladodes from hermaphrodites and females were almost twice as likely to perish due to herbivory by large mammals as those of males. In contrast, cladodes from males tended to perish more frequently due to nonmechanical causes. With regard to the mortality rates due to human actions, no significant differences were detected among the genders (Figure 3). Human‐inflicted sectioning was the less‐frequent action upon cladodes. Remarkably, none of the studied cladodes showed evidence of self‐detaching. Cladodes are firmly attached to the plant, and considerable force has to be applied to detach them, which probably explains why their detachment was achieved only by large herbivores or humans.

### 3.2 Cladode growth rates

**Figure 4:**
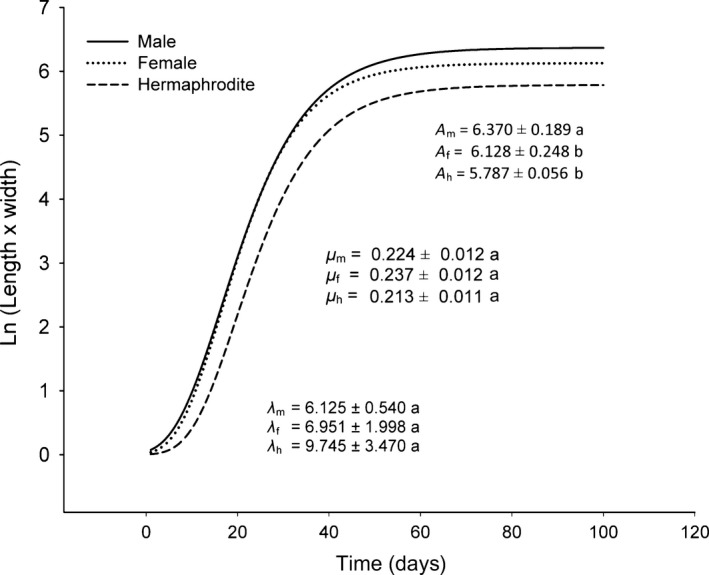
Growth patterns of cladodes of females, hermaphrodites, and males of *Opuntia robusta* in the studied population of Santa Rosa, San Luis Potosí, Mexico. The growth curves were based on the Gompertz model, which gave the best model fit for most of the cladodes analyzed. The parameters of the curves were obtained from the per‐plant average of the three parameters obtained by the fitting of the growth curves of the studied cladodes. The initial lag phase with low growth rates and a time length λ is followed by an exponential phase during which time the maximum growth rate, μ, is attained, at the inflexion point of the growth curve. Then, the growth rate decreases, and the cladode reaches its maximum size A. See text for details. The mean and standard error of the parameters and their pairwise statistical comparisons between the gender type are also shown. Values sharing the same letter are not statistically different (*p* > 0.05)

Cladode growth followed a nonlinear pattern resembling a sigmoidal curve. For most of the studied cladodes, the Gompertz model gave the best fit (87.82%), followed by the logistic model (6.96%) and the nonparametric cubic spline (5.22%). Because we had no control over the timing of bud production in each cladode, the initial sizes of the monitored buds were variable, and we could not always find new cladodes that were small enough to monitor their lag phase. Therefore, this phase was estimated from a subset of cladodes (11 female, 22 hermaphrodite, and 11 male cladodes) that reached the maximum size. Albeit underestimated, our λ could be used for comparative purposes, as our starting sizes were not significantly different among the genders (*F*_2,41_ = 1.05, *p* = 0.36). There were no significant differences among the genders in the length of the lag phase (λ; *F*_2,41_ = 0.54, *p* = 0.60) and maximum growth rates (*F*_2,86_ = 0.83, *p* = 0.44), but maximum cladode sizes differed significantly among the genders (*F*_2,86_ = 6.27, *p* = 0.003) (Figure 4). On average, cladodes from male plants reached larger sizes than cladodes from female and hermaphrodite plants; the latter two were not significantly different (Figure 4). Based on the Gompertz fitting, the exponential growth phase of the cladode was estimated to last approximately 27 day in the three genders. For most cladodes, this phase took place between mid‐May and mid‐July.

### 3.3 Retrogressive growth (shrinkage)

**Figure 5:**
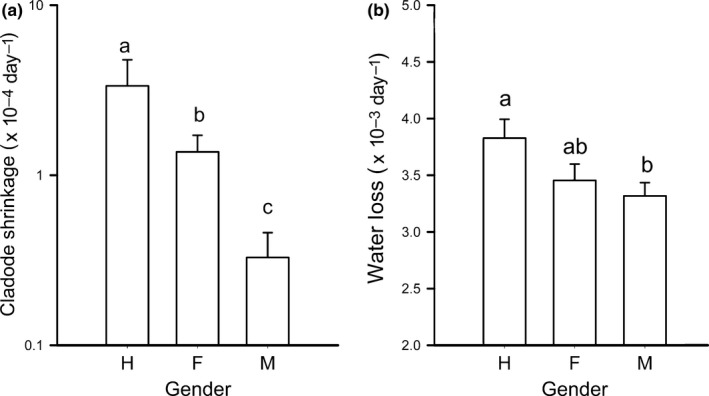
Sexual polymorphism in the rates of shrinkage in cladodes from rooted plants (a), and from artificially detached cladodes (b) of *Opuntia robusta*. Data were obtained from 1‐year‐old cladodes in field conditions, Santa Rosa, San Luis Potosí, Mexico. Values sharing the same letter are not statistically different (*p* > 0.05)

In *O. robusta*, gender polymorphism was associated with contrasting and consistent patterns of shrinkage in cladodes from rooted plants (*F*_2,76_ = 6.33, *p* = 0.003) and in detached cladodes (*F*_2,47_ = 4.37, *p* = 0.018) (Figure 5). Cladodes from hermaphrodite and female rooted plants tended to shrink at similar rates after reaching their maximum size; these rates were significantly different from that of cladodes from males, which shrunk at lower rates. Similarly, the weight of cladodes detached from male plants decreased at a lower rate than the weight of cladodes detached from hermaphrodites, whereas that of cladodes from females was intermediate (Figure 5).

### 3.4 Frequency of reproduction, size to sexual maturation, and heterochrony

**Figure 6:**
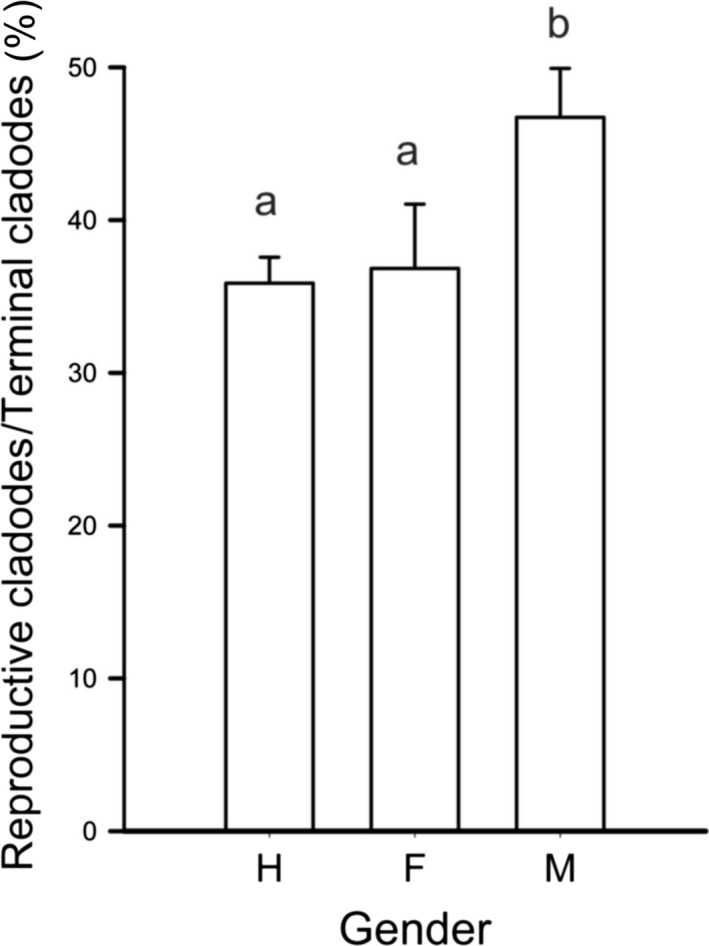
Frequency of flowering cladodes in terminal cladodes of reproductive plants of *Opuntia robusta* in males, females, and hermaphrodites in field conditions. Values sharing the same letter are not statistically different (*p* > 0.05)

The frequency of terminal cladodes with reproductive structures relative to the total number of terminal cladodes (R/T) was significantly different among genders in reproductive individuals (*F*_2,292_ = 5.07, *p* = 0.007). Hermaphrodites and females did not show significant differences in R/T; males, in contrast, displayed significantly higher values of R/T (Figure 6). The reciprocal of the mean of R/T per gender provided an estimate of the average minimum number of terminal cladodes in plants expected to produce a single cladode with reproductive structures, that is, to start flowering. Thus, this value is an indicator of the minimum number of cladodes required for a plant to enter reproductive phase. We estimated that hermaphrodite and female plants with three adult‐size terminal cladodes on average produce one cladode with reproductive structures, while males produce one reproductive cladode with only two adult‐size terminal cladodes per plant. Considering the probability that one young bud produces a mature cladode (Figure 2), females and males require about five and hermaphrodites about eight vegetative budding events to reach the minimum number of terminal cladodes for the production of one reproductive cladode. The production of vegetative buds in *Opuntia* spp. is annual and follows a seasonal pattern similar to that of flowering buds (e.g., Potter, Petersen, & Ueckert, 1986). Thus, the above values indicate that unisexuality accelerates sexual maturation.

### 3.5 Clonality

**Figure 7:**
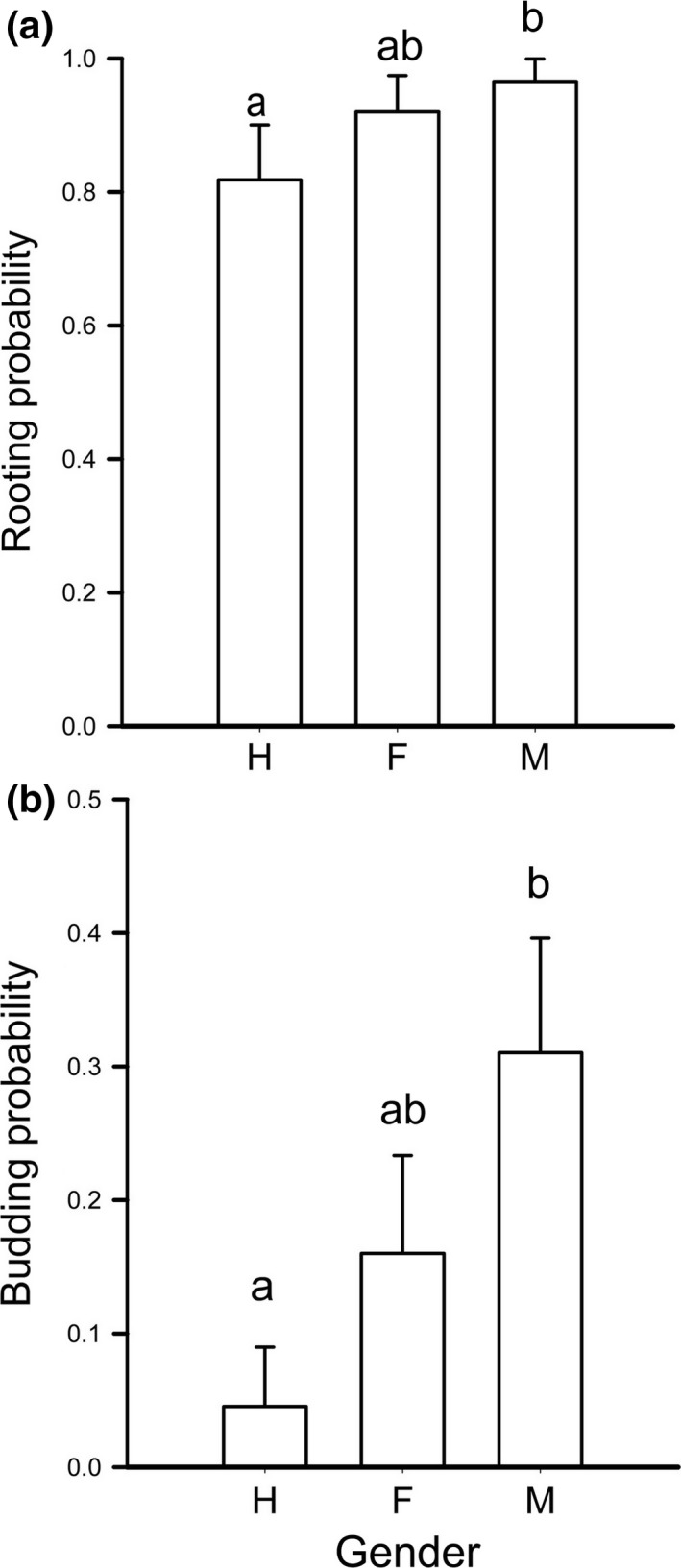
Clonality in *Opuntia robusta* in detached cladodes. (a) Frequency of rooting. (b) Frequency of shooting. Values sharing the same letter are not statistically different (*p* > 0.05)

Detached cladodes from hermaphrodites rooted and produced vegetative buds at lower rates compared with those from males. Females displayed intermediate values of rooting and budding compared with those in males and hermaphrodites (Figure 7).

### 3.6 Combined gender performance in costly demographic options

**Figure 8:**
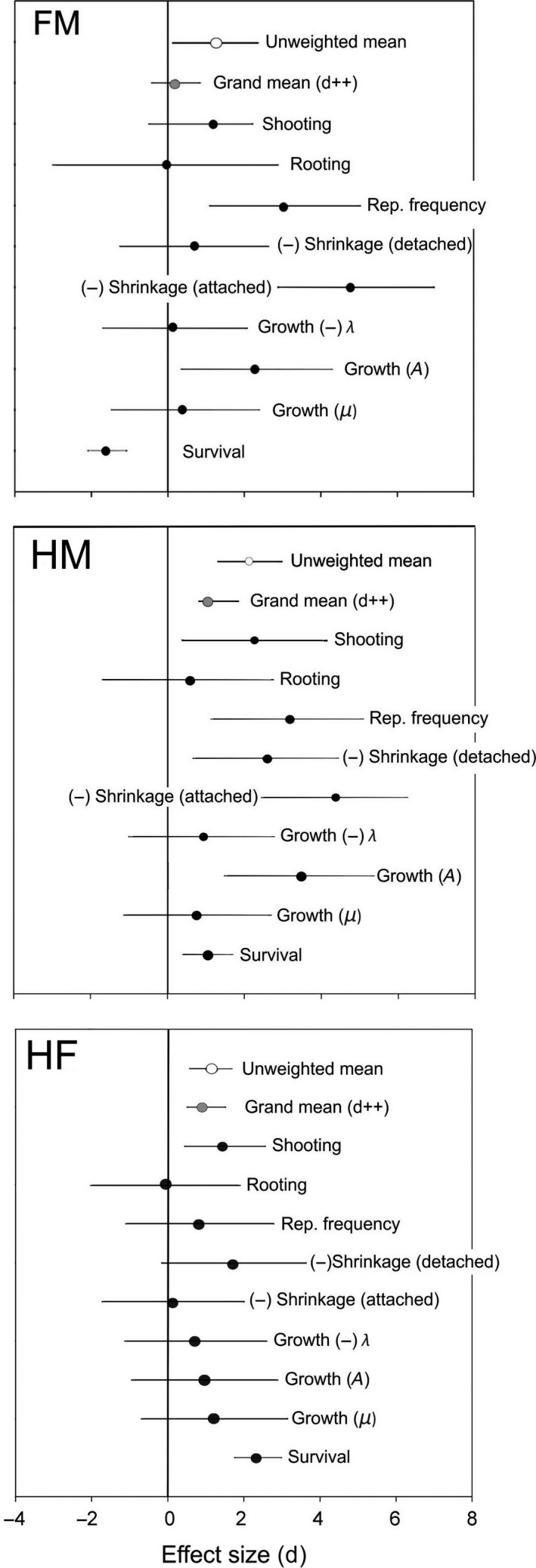
Gender‐associated effect sizes (Hedge's *d*) and 95% bootstrapped confidence intervals for life‐history traits of females and males (FM), hermaphrodites and males (HM), and hermaphrodites and females (HF). Positive effect sizes indicate a higher performance in the investigated trait in the gender that presumably expends less in reproduction relative to the gender that presumably expends more: F < M, H < M, H < F. See text for details and justification. A minus sign (−) for a given trait indicates that the direction of our hypothesis is toward a lower value of the trait in the gender that expends less in reproduction, that is lower shrinkage rates, and a shorter initial lag phase (λ) in cladode growth. The weighted (*d*++) and unweight (*u*) overall effects are also shown

By combining the results of each of the individual tests performed with meta‐analysis techniques and using both weighted and unweighted averages, we detected consistent, strong, and significant effect sizes of gender in life‐history parameters, which were expected to be more costly between males and hermaphrodites (*d*_*++*_ = 1.03, *u *=* *2.11) and between females and hermaphrodites (*d*_*++*_ = 0.93, *u *=* *1.17). Both males and females tended to outperform hermaphrodites in such life‐history options; the latter did not significantly exceed unisexuals in any of the analyzed traits (Figure 8). When comparing the performance of females and males, we detected a strong and significant effect size when using the unweighted average (*u *=* *1.27), but the effect size when using the weighted average was small and not significant (*d*_*++*_ = 0.18; Figure 8). This difference might be explained by the relative small variance in cladode survival and by the gender effect size (*d *=* *−1.59) in the direction opposite to our predictions between males and females. Thus, cladode survival might counterbalance the positive effects of other traits to a greater extent when using the variance‐sensitive weighted average than when using the unweighted average. The combined *p*‐values obtained for the three hypotheses tested were highly significant (*p* < 0.00001) in all cases.

## 4 DISCUSSION

Differences in quantity and quality of the progeny are the most obvious consequences of mating systems and sexual polymorphism, but they are not the only ones. Here, we have shown important differences among *O. robusta* males, females, and hermaphrodites in life‐history parameters. Trade‐offs between reproductive and vegetative functions may account for such differences, as the meta‐analysis revealed that unisexuals tend to exceed hermaphrodites in demographic parameters expected to be more costly. Furthermore, hermaphrodites failed to outperform unisexuals in any trait. The advantages observed of unisexuals may enhance their reproductive potential in the long run and their current clonality potential and therefore could explain the observed increase of unisexuality in the population. To our knowledge, this study is the first to analyze life‐history traits considered essential for plant performance in co‐occurring males, females, and hermaphrodites in natural conditions. Below, we discuss these individual findings and their possible implications for the evolution of unisexuality.

### 4.1 Cladode survival and causes of cladode mortality

Survival of cladodes was higher in females than in hermaphrodites and males, but similar in the latter two. Due to presumably higher reproduction costs in females than in males (see above for a full discussion), survival was expected to be lower in the former than in the latter. However, other vegetative differences among genders may have offset the expected differences. The high vulnerability of cladodes from females to large herbivores might be caused by their lower spine production compared to that of hermaphrodites (Janczur et al., 2014) and males, whose cladodes are usually the most spiny (R. F. del Castillo, *unpublished data*). Spines are deterrent to large herbivores and their ingestion may cause severe injuries (Mellink & Riojas‐López, 2002). Thus, the cladodes of females are probably less protected against large herbivores. However, their higher survival rate suggests that unexplored vegetative differences among the three genders might favor the cladodes of females.

Our results showing that cladodes of males are less likely to perish due to large herbivores compared with those of females and hermaphrodites contrast with the results obtained for other sexually polymorphic species in which males tend to be more vulnerable to herbivory (for reviews see Ågren, Danell, Elmqvist, Ericson, & Hjältén, 1999; Cornelissen & Stiling, 2005; Spigler & Ashman, 2012). Still, this common pattern appears to be true for other kinds of herbivory in *O. robusta*, as cladodes from males were almost twice as likely to perish intact as those of females. This might be caused by sap‐sucking insects, which do not remove parts or the entire cladode and are not deterred by spines (see Janczur et al., 2014). *Opuntia* harbors a highly specialized community of phytophagous insects (Moran, 1980) that reduces survival and reproduction (Miller, Louda, Rose, & Eckberg, 2009). The exact fraction of intact cladodes that die by insect phytophagy and whether their death results from secondary infections in the wounds left by insects warrants further investigation. The difference observed among genders in their vulnerability to herbivory suggests that the defense mechanism against particular kinds of herbivores is differently expressed among genders, making it difficult to generalize the patterns (see also Janczur et al., 2014). Such diversity of responses may provide an additional factor that contributes to the maintenance of sexual polymorphisms.

### 4.2 Cladode growth rates

Our study shows the importance of analyzing the three parameters that define the growth curve in plants with determinate growth. The highest maximum cladode size observed in males could be explained by their lower reproductive costs. However, maximum growth rates and lag times were not significantly different among genders. Males may not attain the expected higher maximum growth rates because of their higher spine production. The different patterns of spine production among genders may blur the expected differences in growth. Our results contrast with the commonly higher growth rates found in males compared to females (see Barrett & Hough, 2013). However, we are not aware of any other study considering the parameters of the growth curve.

### 4.3 Clonality

The product of the estimated shooting (rooting and budding) probability in detached cladodes (Figure 7) and the probability that the newly produced cladode reaches its final size (Figure 2) provides an estimate of clonality. With such information, we estimate that males and females were 8.35‐ and 5.25‐fold, respectively, more likely to produce a new plant from detached cladodes than hermaphrodites. The reproductive advantages of unisexuality are hindered by limited pollination services in some populations (del Castillo & Trujillo‐Argueta, 2009). Thus, alternative ways of propagation are likely strongly selected. Clonality appears to be an alternative and convenient way of propagation for plant species with breeding systems that depend on pollinators (e.g., Vallejo‐Marin & O'Brien, 2006). Accordingly, the higher clonality of unisexuals compared to hermaphrodites provides the most plausible explanation for the maintenance and increase of unisexuality in *O. robusta*. Because of pollen limitation and low germination rates, sexual reproduction can be less effective for plant recruitment than clonal propagation in *Opuntia* (Pimienta Barrios & del Castillo, 2002; Reyes‐Agüero, Aguirre‐Rivera, & Valiente‐Banuet, 2006).

Since self‐detachment is unlikely in *O. robusta*, the advantages of high clonality are only effective in habitats with extrinsic sources of stem sectioning, such as those inflicted by ranchers. Large domestic or naturalized mammals such as cattle or donkeys usually consume wild cactus and, to get access to spineless parts of the plant, they kick, uproot, or detach cactus parts (Hicks & Mauchamp, 1995; Jiménez‐Sierra & Eguiarte, 2010; Lindsay, 1955). To our knowledge, humans and large domestic animals are the only ones capable of detaching the heavy and well‐attached cladodes of *O. robusta* and thereby inducing cloning. However, in the Late Pleistocene, large extinct mammals, including *Equus* spp. (horses) and *Mammuthus* spp. (mammoths) (Pérez‐Crespo et al., 2009), inhabited the semiarid dry lands of Central Mexico and they might have played a similar role to that of the extant large introduced mammals in *Opuntia*‐dominated scrubs (Janzen, 1986).

### 4.4 Retrogressive growth (shrinkage)

Shrinkage of cactus can be explained by a negative carbon balance through depletion of carbohydrates during stress conditions (e.g., Holthe & Szarek, 1985), but mostly by transpiration (Nobel, 1988). Hence, different shrinkage rates may imply different levels of water vapor conductance. Cactus may expend considerable energy in mechanisms that reduce transpiration, such as the production of hydrophobic, thick, and waxy cuticles (see Terrazas‐Salgado & Mauseth, 2002). Thus, allocating a low energy budget to reproduction probably implies that more resources might be used for reducing water loss. In agreement with this hypothesis, cladodes detached or attached to hermaphroditic mother plants shrunk at the highest rates, and cladodes from females shrunk at intermediate rates between cladodes from males and hermaphrodites. In other species of *Opuntia*, up to 55% of the plant size may be reduced due to shrinkage (*O. macrorhiza*, 2011), which has been associated with low rainfall levels (Mandujano, Montaña, Franco, Golubov, & Flores Martínez, 2001).

Lower shrinkage rates likely imply more resources for other vegetative functions. Daughter cladodes compete with mother cladodes for water, hastening the effects of drought (Pimienta‐Barrios, Zañudo‐Hernández, Rosas‐Espinosa, Valenzuela‐Tapia, & Nobel, 2005). In addition, transforming a detached cladode into a new plant depends on the cladode's water content (Mondragón‐Jacobo, Méndez‐Gallegos, & Olmos‐Oropeza, 2003). Thus, in detached and prerooting cladodes, water content is even more crucial as water losses cannot be replenished by root absorption. Thus, under water stress, detached cladodes of *O. robusta* curtail the generation of new roots and cladodes (Pimienta‐Barrios, del Castillo‐Aranda, & Nobel, 2002). This might explain why hermaphrodites, which present the highest shrinkage rates, displayed the lowest probabilities of cloning and cladode generation.

Shrinkage is not necessarily maladaptive: it is associated with a higher recovery speed after disturbance in herbaceous perennials (Salguero‐Gómez & Casper, 2010). In *Opuntia* spp., it may function as a cryoprotection mechanism by reducing protoplast decrement and desiccation under freezing temperatures (Koch & Kennedy, 1980; Littlejohn & Williams, 1983; Nobel & Bobich, 2002). By displaying the highest shrinkage, hermaphrodites might reduce clonality, growth, and drought tolerance but increase frost tolerance. Thus, the impact of shrinkage on fitness appears to be habitat contingent in *O. robusta*. To our knowledge, our study is the first to report gender‐associated differences in shrinkage.

### 4.5 Life‐history traits and reproductive costs

The differences in growth, survival, flowering frequency, and cloning observed among genders might be attributed, at least in part, to differences in reproductive allocation. Higher allocation to reproduction may imply lower resources for other competing plant functions. Thus, as commonly observed in dioecious populations (see Delph, 1999), the relative performance of genders in life‐history traits within trioecious populations can be largely deduced by inspecting differences in their reproductive costs.

The higher frequency of flowering in males than in hermaphrodites and the earlier reproductive maturation of unisexuals compared to hermaphrodites can be explained by the higher costs of reproduction incurred by hermaphrodites followed by females and males. A higher investment in reproduction in a given season likely compromises the production of reproductive structures in the following year. This should be more evident when reproductive expenses are high, as is the case of hermaphrodites. Manipulative experiments altering plants' reproductive effort by treatments such as pollen supplementation, might provide a direct test of this hypothesis (see Alvarez‐Cansino, Zunzunegui, Barradas, & Esquivias, 2010 for an example in dioecious species).

For long‐lived perennials, as *O. robusta*, saving resources in reproduction in one reproductive bout can be beneficial in the long run. With pollen limitation, increases in allocation of resources in gamete production in a given time may follow a law of diminishing returns (a decelerating curve). Instead, allocating more resources to survival and growth by reducing reproduction in a given season could enhance ramet production, survival, and growth. This means a higher photosynthetic surface, and, thus, increases in clonality and reproductive potential in the future (see Pan & Price, 2002). Furthermore, since growth is inherently exponential, such increases may be also exponential (see Seger & Eckhart, 1996). Finally, redistributing flower production in more ramets and during several seasons may decrease the rates of pollen discounting and enhance pollination quality (see Harder & Barrett, 1995; Liao & Harder 2014, Van Drunen et al. 2015). Clearly for long‐living perennials, evaluating reproductive potential based on a single reproductive bout may be misleading. Here we show that unisexuals are more precocious than hermaphrodites and display a higher incidence of reproduction among ramets. Estimating the fitness of the three morphs would require in addition to the information we have now available, knowing the number of years each morph can become reproductive and the conditions they have experienced through such time.

### 4.6 Unisexuality and life‐history trade‐offs

Life‐history theory postulates the impossibility of simultaneous increases in competing demographic traits, leading to the classical trade‐offs that characterize life‐history strategies (Stearns, 1992). However, unisexuals can display simultaneous increases in antagonistic demographic options relative to those observed in hermaphrodites. The age at sexual maturity correlates negatively with the probability of transitioning to larger plant sizes in the so‐called fast–slow axis (Salguero‐Gomez et al., 2016). In *O. robusta*, males, females, or both may exhibit higher survival probabilities and may start to reproduce earlier than hermaphrodites. Thus, unisexuality may blur the classically observed trade‐offs in life‐history traits, probably due to their lower energy allocation to reproduction compared to that of hermaphrodites. Under less‐stressful conditions, in which resources are not very limited, such kind of trade‐offs usually becomes undetectable (Agrawal, Conner, & Rasmann, 2010; van Noordwijk & de Jong, 1986).

### 4.7 Life‐history strategies involve both trade‐offs and epigenetic changes

Unisexuals outperformed hermaphrodites in their capability to generate new plants from sectioned stems. This capability arises from the plasticity of cladode stem cells to undertake different developmental pathways following the detection of organismic (cladode detachment from the mother plant and areole position) and environmental (probably light) cues. All cladodes used for exploring clonality were terminal cladodes detached from sexual plants. In rooted plants, at least 40% of these cladodes should have developed flowering buds (Figure 6) but none did. Hence, severing appears to modify the ontogeny at stem cell level by inhibiting the development of flowering buds and activating specific developmental pathways on a selected group of areoles at selected cladode sections; namely, (a) developing vegetative buds on one or two areoles on the upper side and near the apex of the detached cladode; (b) inducing adventitious rooting in some areoles on the downward side of the cladode; and (c) resetting of flowering, since flowering buds are only produced after a minimum number of cladodes has been produced (see above). Because of such fine‐scale epigenetic decisions, severing transforms terminal cladodes of reproductive plants from a sexual reproductive system into a cloning organ. Without epigenetic reprogramming, cloning would be impossible.

The acceleration of reproductive maturation (progenesis) is another ontogenetic consequence of unisexuality. Compared to that of hermaphrodites, the stem cells of unisexuals generate flowers in smaller plants and after a lower number of budding events. Progenesis has long been recognized as one of the easiest and fastest pathways to boost population growth (Cole, 1954). In *O. robusta*, limited pollinator services may offset these advantages, at least in the Santa Rosa population. However, progenesis can be crucial in situations where pollination services are less limited, as those expected in high‐density populations where dioecy prevails (del Castillo, 1986). In such situations, unisexuals can attain their full reproductive potential and reproductively outperform hermaphrodites. In summary, the performance in life‐history traits detected in unisexuals does not appear to depend exclusively on reproduction savings but also on important ontogenetic adjustments, allowing unisexuals to reproduce earlier or produce more shoots in detached cladodes compared to hermaphrodites.

## 5 CONCLUSIONS

Our study detected consistent patterns in life‐history traits among *O. robusta* genders, from which we can extract the following lessons: (a) Gender may have a significant influence on life‐history traits relevant for fitness. (b) Unisexuals may display simultaneous increases in life‐history traits known to compete for the same limiting resources. (c) For presumably resource‐costly options in life‐history traits, unisexuals may outperform hermaphrodites, particularly if their reproductive expenditure is lower than that of hermaphrodites, as is probably the case of female plants under pollen‐limited conditions. (d) Gender‐associated life‐history traits may contribute to the maintenance of separated sexes, but only under particular circumstances, sometimes of anthropogenic origin. (e) In plants with determinate growth, growth cannot be described properly based solely on growth rates and assuming a linear or log‐linear pattern. Logistic fitting of the growth curve and considering shrinkage are essential. (f) Life‐history strategies may depend on fine stem cell programming and reprogramming following organismic and environmental cues. Overall (g), the ecology, evolution, and epigenetics of gender‐polymorphic species deserve more attention; (h) sexual dimorphism in plants may not be as well developed as in animals (Barrett & Hough, 2013), but this study shows evidence that traits little or not studied, such as shrinkage may contribute to plant sexual polymorphism. (i) The evolution of sexual polymorphisms and breeding system cannot be fully understood without considering reproductive and vegetative consequences and their interactions.

## CONFLICT OF INTEREST

None declared.

## AUTHOR CONTRIBUTIONS

RFDC conceived the study, did the field work, performed the statistical analyses and data interpretation, and wrote the manuscript. STA participated in the design of the study, assisted in field work, and helped in data interpretation.

## DATA AVAILABILITY

Dryad: https://doi.org/10.5061/dryad.k658bh2
